# Thromboelastography as an early prediction method for hypofibrinogenemia in emergency department patients with primary postpartum hemorrhage

**DOI:** 10.1186/s13049-024-01263-5

**Published:** 2024-09-13

**Authors:** Sang-Min Kim, Chang Hwan Sohn, Hyojeong Kwon, Seung Mok Ryoo, Shin Ahn, Dong Woo Seo, Won Young Kim

**Affiliations:** Department of Emergency Medicine, Asan Medical Center, University of Ulsan College of Medicine, Seoul, Republic of Korea

**Keywords:** Postpartum hemorrhage, Blood transfusion, Blood coagulation tests, Fibrinogen, Predictive value of tests

## Abstract

**Background:**

Timely and accurate assessment of coagulopathy is crucial for the management of primary postpartum hemorrhage (PPH). Thromboelastography (TEG) provides a comprehensive assessment of coagulation status and is useful for guiding the treatment of hemorrhagic events in various diseases. This study aimed to evaluate the role of TEG in predicting hypofibrinogenemia in emergency department (ED) patients with primary PPH.

**Methods:**

We conducted a retrospective observational study in the ED of a university-affiliated tertiary hospital between November 2015 and August 2023. TEG was performed upon admission. The cutoff value for hypofibrinogenemia was 200 mg/dL. The primary outcome was the presence of hypofibrinogenemia.

**Results:**

Among the 174 patients, 73 (42.0%) had hypofibrinogenemia. The need for massive transfusion was higher in the hypofibrinogenemia group (37.0% vs. 5.0%, *p* < 0.001). Among the TEG parameters, all values were significantly different between the groups, except for lysis after 30 min, suggesting a tendency toward hypocoagulability. Multivariable analysis revealed that the alpha angle (odds ratio (OR) 0.924, 95% confidence interval (CI) 0.876–0.978) and maximum amplitude (MA) (OR 0.867, 95% CI 0.801–0.938) were independently associated with hypofibrinogenemia. The optimal cutoff values for the alpha angle and maximum amplitude (MA) for hypofibrinogenemia were 63.8 degrees and 56.1 mm, respectively.

**Conclusion:**

Point-of-care TEG could be a valuable tool for the early identification of hypofibrinogenemia in ED patients with primary PPH.

## Background

Postpartum hemorrhage (PPH) is the leading cause of maternal death worldwide [1]. Prompt blood transfusion and treatment for coagulopathy are crucial for improving outcomes in patients with massive hemorrhage [2]. A recent statement on the management of PPH recommends using coagulation tests to guide the appropriate utilization of blood components [3].

Hypofibrinogenemia (fibrinogen level < 200 mg/dL) is a predictor of massive bleeding, with a positive predictive value of 75–100% [4, 5]. Additionally, a fibrinogen level greater than 200 mg/dL is an appropriate target for achieving hemostasis [6]. Early identification and treatment of coagulopathy are crucial in patients with PPH, although accurately predicting this condition at the initial stages is challenging. During PPH, the concentration of fibrinogen decreases to a critically low level before the levels of other clotting factors [7]. In a previous study, we confirmed that hypofibrinogenemia was associated with the need for massive transfusion in patients with primary PPH referred to the emergency department (ED) after delivery at other hospitals or clinics [8]. However, considering that the turnaround time for fibrinogen level measurement is approximately 1 h or more, it would be very clinically beneficial if a method could be used to predict hypofibrinogenemia early in the patient's visit [9].

Thromboelastography (TEG) is a rapid point-of-care test that assesses the entire process of blood clot formation, including clot initiation, progression, termination, and fibrinolysis [10]. TEG provides faster results than conventional coagulation tests and is particularly valuable in guiding hemostatic therapy in trauma, liver transplantation, and other surgeries [11–13]. The utilization of TEG in the postpartum phase has been shown to reduce both hemorrhage and the need for blood transfusions [14, 15]. Furthermore, as the incidence of hypofibrinogenemia increases with the severity of bleeding [16, 17], it is crucial to promptly identify hypofibrinogenemia in patients presenting with PPH in the emergency department (ED). We hypothesized that TEG would accurately predict hypofibrinogenemia based on fibrinogen levels measured by conventional methods. Although there have been prior studies examining the use of TEG to predict hypofibrinogenemia, there is a lack of research specifically focused on patients with PPH who visited the ED.

This study aimed to assess the use of TEG as an early predictor of hypofibrinogenemia in ED patients with primary PPH.

## Methods

### Study design and setting

This was a retrospective observational study conducted in the ED of a 2800-bed, university-affiliated tertiary referral center in South Korea from November 2015 to August 2023. The study included all patients with primary PPH who were referred to the ED and underwent TEG upon ED admission. Primary PPH was defined as hemorrhage occurring within 24 h of delivery, requiring fluid resuscitation or transfusion [18]. Patients referred for evaluation and management of PPH from other hospitals or obstetric clinics after delivery were also included [18]. Patients with inappropriate initial TEG data were excluded from the study. The primary outcome of this study was the presence of hypofibrinogenemia, with a cutoff value of 200 mg/dL, identified as a predictor of PPH severity in previous studies [4, 19].

### Data collection

The baseline and clinical characteristics of the patients were extracted from electronic medical records. These characteristics included age, parity, type of delivery, initial mental status, initial vital signs, initial laboratory findings, amount of blood transfusion, need for massive transfusion (MT), and clinical outcomes such as embolization, hysterectomy, length of hospital stay, admission to the intensive care unit (ICU), and in-hospital death. MT was defined as the transfusion of 10 units or more of packed red blood cells within the first 24 h after the onset of PPH [18]. We determined the MT by calculating the total volume of blood transfused at the hospital and prehospital levels, including the ICU, general ward, and ED. The patient’s initial mental status was assessed using the AVPU scale (Alert/Verbal/Painful/Unresponsive) during the triage stage upon their arrival at the ED. Initial vital signs, such as systolic blood pressure, diastolic blood pressure, heart rate, and body temperature, were also measured during the triage stage. The initial shock index was calculated based on the initial vital signs. Emergency physicians or obstetricians caring for patients with primary PPH determined whether a blood transfusion should be performed, as well as the quantity and type of blood to be transfused. The data collection was conducted by two emergency physicians using a pre-drafted data abstraction form. The completion and accuracy of each data abstraction form were verified by one of the two emergency physicians.

### Thromboelastography

Upon admission to the ED, a TEG test was conducted. The attending nurse drew approximately 4 mL of whole blood into vials containing citrate. The TEG analysis began in real-time once the blood samples reached the laboratory. For the standard TEG analysis, the whole blood was mixed with kaolin in a vial and then placed into the TEG cup pre-filled with CaCl_2_ for recalcification. The TEG analysis was carried out by laboratory technicians using a computerized coagulation analyzer (Model 5000; Hemonetics Corporation, Boston, MA). The TEG analyzer monitored the dynamic changes in the samples as the cups oscillated and rotated, simultaneously measuring the physical properties of the clot. The recorded variables included reaction time (R, measured in minutes, indicating the rate of initial fibrin formation), clot formation speed (K, measured in minutes, indicating the clot growth kinetics), alpha angle (α, reflecting the clot growth kinetics), maximum amplitude (MA, measured in mm, indicating the clot strength), and lysis after 30 min (LY30, measured in %, representing the proportional reduction in amplitude after MA, indicating fibrinolysis). The TEG software (TEG Analytic Software 4.2.3; Hemonetics Corporation) was used for the analyses, and all tests were conducted and analyzed following the manufacturer’s instructions.

### Statistical analysis

Continuous variables with normal distributions were represented as the mean ± standard deviation, while those with non-normal distributions were represented using the median and interquartile range (IQR). Categorical variables were presented as numbers and percentages. The Student’s t-test or the Wilcoxon rank-sum test was used for continuous variables, while the chi-square test or Fisher’s exact test was used for categorical variables. TEG analysis involved calculating the area under the receiver operating characteristic (ROC) curve, also known as the area under the curve (AUC). Cut-off values for TEG parameters, such as K, the alpha angle, and MA, which showed high discriminatory power, were determined based on an AUC greater than 0.750. The optimal cut-off value was the point that maximized the sum of sensitivity and specificity − 1. Multivariable logistic regression analysis was conducted to identify independent factors associated with hypofibrinogenemia. Variables associated with hypofibrinogenemia in the univariable analysis (*p* < 0.1) were included in the logistic regression analysis. After confirming multicollinearity through linear regression, we excluded variables with multicollinearity. Specifically, we removed certain laboratory values that can be obtained concurrently with fibrinogen at different time frames. We conducted multivariable analysis using the backward stepwise regression process. The goodness of fit of the logistic model was assessed using the Hosmer–Lemeshow test. The results of the multivariable logistic regression analysis were reported as odds ratios (ORs) and 95% confidence intervals (CIs). In addition, standard statistical methods were used to calculate the sensitivity, specificity, positive predictive value (PPV), and negative predictive value (NPV). A two-sided *p* value of less than 0.05 was considered to indicate statistical significance. The statistical analyses were performed using PASW Statistics for Windows Version 23.0 (IBM Corp., Armonk, NY, USA).

## Results

### Baseline and clinical characteristics

**Fig. 1:**
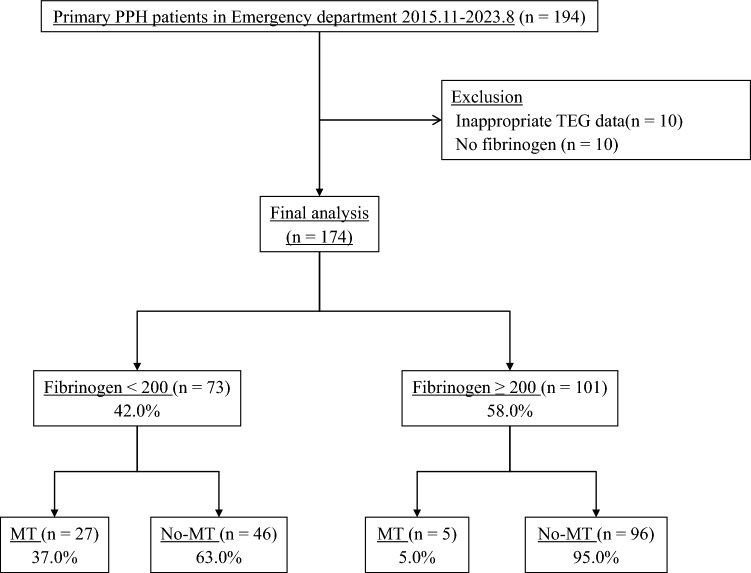
Patient flow diagram. This figure was produced with the assistance of Microsoft Power Point 2016 (Microsoft Ltd, Washington, United States). TEG; thromboelastography, MT; massive transfusion

A total of 194 patients with primary PPH were enrolled in the study. We excluded 10 patients from the analysis because their TEG data were not suitable, as they did not include the complete TEG values. We also excluded 10 patients who did not undergo fibrinogen assays. Of the 174 patients included, 73 (42.0%) had a fibrinogen level lower than 200 mg/dL (Fig. 1).

Table 1 shows the baseline and clinical characteristics of patients categorized based on the presence of hypofibrinogenemia. There were no significant differences in age, parity, delivery type, or mental status between the two groups. Among the vital signs, the initial systolic and diastolic blood pressures were lower in the hypofibrinogenemia group. The initial shock index was higher in the hypofibrinogenemia group (0.9 [0.7–1.1] vs. 0.8 [0.7–0.9], *p* = 0.003).

**Table 1:** Comparison of baseline and clinical characteristics based on the presence of hypofibrinogenemia in patients with primary postpartum hemorrhage

Variables	Hypofibrinogenemia group (n = 73)	No-hypofibrinogenemia group (n = 101)	*p* value
Age, years	33.8 ± 4.1	32.6 ± 4.0	0.067
Parity			0.345
Primipara	41 (56.2)	65 (64.4)	
Multipara	32 (43.8)	36 (35.6)	
Delivery type			0.345
Vaginal delivery	41 (56.2)	65 (64.4)	
Cesarean section	32 (43.8)	36 (35.6)	
Mentality			0.316
Alert	68 (93.2)	99 (98.0)	
Verbal	3 (4.1)	2 (2.0)	
Painful	1 (1.4)	0 (0.0)	
Unresponsive	1 (1.4)	0 (0.0)	
Initial vital signs			
Systolic blood pressure, mmHg	109 (93–123)	117 (107–133)	0.004
Diastolic blood pressure, mmHg	67 (58–79)	76 (65–84)	0.008
Pulse rate, per min	97 (85–113)	90 (80–105)	0.079
Respiratory rate, per min	20 (18–20)	20 (18–20)	0.097
Body temperature, °C	37.5 (36.9–38.0)	37.4 (36.9–37.8)	0.346
Shock index	0.9 (0.7–1.1)	0.8 (0.7–0.9)	0.003

Values are expressed as the mean ± standard deviation, median [interquartile range], or number (%)

The initial laboratory findings, amount of blood transfusion, and clinical outcomes are shown in Table 2. The initial lactate levels did not show significant differences between the two groups. Hemoglobin, hematocrit, and platelet levels were significantly lower in the hypofibrinogenemia group. Additionally, prothrombin time (International Normalized Ratio), fibrin degradation product, and d-dimer levels were significantly higher in the hypofibrinogenemia group.

**Table 2:** Comparison of initial laboratory findings, amount of blood transfusion, and clinical outcomes based on the presence of hypofibrinogenemia in patients with primary postpartum hemorrhage

Variables	Hypofibrinogenemia group (n = 73)	No-hypofibrinogenemia group (n = 101)	*p* value
Laboratory findings			
Lactate, mmol/L	2.3 (1.8–3.2)	2.2 (1.6–3.0)	0.138
Hemoglobin, g/dL	8.7 (7.8–10.1)	10.9 (9.5–12.3)	< 0.001
Hematocrit, %	27.6 (23.6–31.2)	32.3 (29.8–36.7)	< 0.001
Platelet, × 10^3^/uL	125 (103–146)	175 (148–214)	< 0.001
PT (INR)	1.3 (1.2–1.6)	1.1 (1.0–1.1)	< 0.001
FDP, ug/mL	120 (57–125)	34 (17–82)	< 0.001
D-dimer, ug/mL	35.2 (11.8–35.5)	10.9 (5.8–25.4)	< 0.001
Amount of blood transfusion (units)			
Packed red blood cells	6.0 (4.0–11.0)	3.0 (1.0–5.0)	< 0.001
Fresh frozen plasma	5.0 (3.0–10.0)	1.0 (0.0–4.0)	< 0.001
Platelet concentrate	0.0 (0.0–8.0)	0.0 (0.0–0.0)	< 0.001
Cryoprecipitate	0.0 (0.0–3.0)	0.0 (0.0–0.0)	0.008
Clinical outcome			
Massive transfusion	27 (37.0)	5 (5.0)	< 0.001
Embolization	50 (68.5)	37 (36.6)	< 0.001
Hysterectomy	1 (1.4)	1 (1.0)	1.000
Length of hospital stay, days	2.0 (2.0–4.0)	2.0 (1.0–3.0)	0.003
ICU admission	2 (2.7)	5 (5.0)	0.700
In-hospital death	1 (1.4)	0 (0.0)	0.420

Values are expressed as the median [interquartile range] or number (%)

PT, prothrombin time; INR, international normalized ratio; FDP, fibrinogen degradation production; ICU, intensive care unit

The proportion of patients receiving MT was significantly greater in the hypofibrinogenemia group (37.0% vs. 5.0%, *p* < 0.001). Embolization (68.5% vs. 36.6%, *p* < 0.001) was more frequently conducted in the hypofibrinogenemia group. The length of hospital stay was shorter in the non-hypofibrinogenemia group. One patient with hypofibrinogenemia died during her hospital stay.

### Thromboelastography analysis

Table 3 shows the TEG parameters for each group. The hypofibrinogenemia group had higher levels of R and K compared to the non-hypofibrinogenemia group. Additionally, the hypofibrinogenemia group had lower alpha angle and MA levels. Among the TEG values, the K, MA, and alpha angle values were found to be discriminative, as defined by an AUC > 0.750. The optimal cutoff values for predicting the need for hypofibrinogenemia using TEG were K > 1.9 min, alpha angle < 63 degrees, and MA < 60 mm.

**Table 3:** Comparison of thromboelastographic parameters based on the presence of hypofibrinogenemia in patients with primary postpartum hemorrhage

Variables	Hypofibrinogenemia group (n = 73)	No-hypofibrinogenemia group (n = 101)	AUC	*p* value
R, minutes	4.5 (3.5–5.5)	3.7 (3.2–4.1)	0.710	< 0.001
K, minutes	3.1 (2.3–5.1)	1.3 (1.1–1.7)	0.872	< 0.001
K > 1.9 min	57 (78.1)	15 (14.9)		< 0.001
Alpha angle, degrees	51.2 (39.9–61.3)	70.7 (65.3–74.1)	0.861	< 0.001
Alpha angle < 63 degrees	60 (82.2)	18 (17.8)		< 0.001
MA, mm	51.8 (42.2–59.6)	66.2 (61.2–70.3)	0.885	< 0.001
MA < 60 mm	55 (75.3)	21 (20.8)		< 0.001
LY30, %	0.0 (0.0–0.3)	0.0 (0.0–0.7)	0.528	0.478

Values are expressed as the median [interquartile range] or number (%)

AUC, area under the receiver operating characteristics curve; R, reaction time; K, kinetic time; MA, maximum amplitude; LY30, lysis after 30 min

### Factors predicting the need for massive transfusion

Multivariable analysis was conducted to identify independent factors associated with the presence of hypofibrinogenemia. The initial variables included lactate level, systolic blood pressure, diastolic blood pressure, shock index, and TEG values (Table 4). The multivariable regression analysis revealed three clinical factors that were independently associated with the presence of hypofibrinogenemia: initial systolic blood pressure (OR 0.970, 95% CI 0.949–0.990; *p* = 0.004), alpha angle (OR 0.913, 95% CI 0.863–0.966; *p* = 0.002), and MA (OR 0.885, 95% CI 0.819–0.956; *p* = 0.002).

**Table 4:** Multivariable analysis for predicting hypofibrinogenemia in patients with primary postpartum hemorrhage

Variables	Multivariate OR [95% CI]		*p* value
	Adjusted OR	95% CI	
Initial SBP (every 1 mmHg increase)	0.970	0.949–0.990	0.004
Alpha angle (every 1-degree increase)	0.913	0.863–0.966	0.002
MA (every 1 mm increase)	0.885	0.819–0.956	0.002

Multivariable analysis included logistic regression analysis and backward elimination

OR, odds ratio; CI, confidential interval; SBP, systolic blood pressure; MA, maximum amplitude

### ROC plots and test performance of TEG parameters for predicting hypofibrinogenemia

**Fig. 2:**
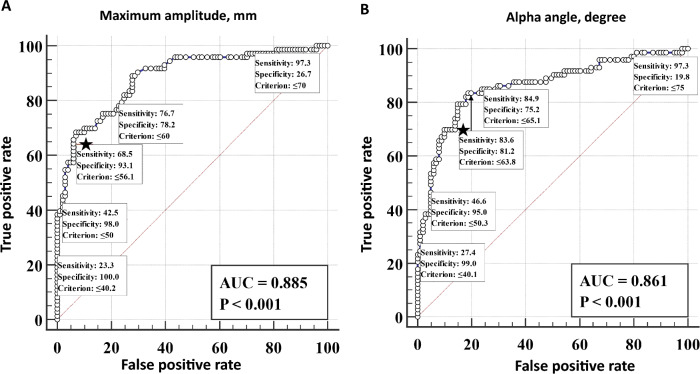
ROC plots of TEG values for predicting the presence of hypofibrinogenemia in postpartum hemorrhage patients. **A** Maximal amplitude. **B** Alpha angle. *The star icon indicates the optimal cutoff value

Figure 2 shows ROC plots of TEG values for predicting the presence of hypofibrinogenemia in PPH patients. The optimal cut-off values for maximizing the sum of sensitivity and specificity—1 were identified as an Alpha angle of < 63.8 degrees and MA of < 56.1 mm. For clinical utility, various potential cut-off values for TEG parameters for hypofibrinogenemia were examined, with MA ranging from 40 to 70 and the alpha angle ranging from 40 to 75, respectively. An alpha angle < 63.8 degrees showed a sensitivity of 83.6%, specificity of 81.2%, PPV of 76.3%, and NPV of 87.2%.

## Discussion

In this study, among the 174 ED patients with primary PPH, 73 (42.0%) had hypofibrinogenemia. MT was more frequently performed in the hypofibrinogenemia group. TEG values differed significantly between the groups, indicating a tendency towards hypocoagulability in the hypofibrinogenemia group. According to multivariable analysis, MA and the alpha angle were independently associated with the presence of hypofibrinogenemia. The optimal cutoff values for predicting hypofibrinogenemia were 56.1 mm for MA and 63.8 degrees for the alpha angle.

Fibrinogen levels below 200 mg/dL were strongly associated with progression toward severe PPH [20]. However, the incidence of hypofibrinogenemia varies greatly among studies. In a Danish randomized study with 224 severe PPH patients, only 5 had a fibrinogen concentration lower than 200 mg/dL [21]. In another trial with 663 PPH patients, 55 patients with a FIBTEM value of 15 mm (equivalent to a fibrinogen value of 300 mg/dL) were randomly assigned to receive either fibrinogen concentrate or a placebo [6]. They observed no improvement in outcomes, and only 7 patients developed a fibrinogen level less than 200 mg/dL. However, in a nationwide cohort study in the Netherlands involving 1,312 severe PPH patients, 342 (26%) had a fibrinogen level lower than 200 mg/dL [22]. Furthermore, they found that women who developed adverse outcomes reached hypofibrinogenemia earlier than those who did not. In this study, we found that 42% of patients developed hypofibrinogenemia, a rate significantly greater than that reported in previous studies. This can be explained by differences in the study populations. Given that fibrinogen levels decrease more rapidly in massive hemorrhages, patients who visit the ED with PPH might have a more severe clinical presentation. Early identification of hypofibrinogenemia is essential for managing ED patients.

It is challenging for physicians to detect coagulopathy in patients with PPH due to its distinct hemostatic characteristics compared to bleeding in other settings, such as trauma [23]. During PPH, conventional coagulation parameters, such as PT, aPTT, and platelet count, do not frequently change despite significant blood loss [7, 24]. We observed significant differences in PT and platelet count between the groups, although the deviation from the normal range was not substantial. This finding is similar to the results of a previous study, where PT > 1.5 times the reference range and platelet count < 75 × 109/L were observed in only 3.4% and 5.1% of PPH patients with blood loss greater than 2500 mL, respectively [17]. TEG may provide advantages in accurately assessing coagulation status, taking into account the dynamic clinical presentation of PPH.

Previous studies have indicated that viscoelastic hemostatic assays, such as TEG and rotational thromboelastometry (ROTEM), can detect hypofibrinogenemia in PPH [25–28]. A previous study involving PPH patients revealed a strong correlation (r > 0.8) between ROTEM Fibtem A5 and fibrinogen levels [25, 26]. Bell et al. reported that ROTEM Fibtem A5 ≤ 11 mm could detect fibrinogen levels ≤ 2 g/dL with a sensitivity of 0.76 and a specificity of 0.96 [27]. Another study involving 521 women reported similar results with TEG 6 s [28]. However, previous studies have reported that the incidence of hypofibrinogenemia is lower than 5% [27, 28]. We observed consistent results in patients with more severe conditions who visited the ED. Furthermore, McNamara et al. found that introducing a TEG-guided protocol for managing patients with PPH resulted in a substantial decrease in the use of blood products and transfusion-related complications [29].

In this study, MT was more frequently performed in the hypofibrinogenemia group. Previous observations indicated that lactate levels and the shock index were independently associated with the need for MT [18, 30]. However, this study showed that lactate levels did not show significant differences between the groups, and the shock index was not an independent predictor of hypofibrinogenemia. This suggests that not only disease severity but also coagulopathy play a critical role in the development of adverse outcomes.

Although recent guidelines emphasize the role of management in coagulopathy, the recommendations for coagulation tests and transfusions are inconsistent [31, 32]. Given the distinct characteristics of hemostasis during pregnancy, TEG could provide valuable insights for the early detection of coagulopathy. We have proposed several potential thresholds for predicting hypofibrinogenemia for clinical application. Further study is needed to determine the significance of TEG in the management of PPH.

This study has several limitations. First, the study design used was retrospective, which could introduce biases and confounding variables that may impact the validity of our findings. Second, the findings may not be generalizable because the study was conducted at a single center with patients visiting the ED. It might not be appropriate to apply our findings to patients in hospital delivery units, obstetric clinics, or other healthcare facilities. Third, during the study period, the MT protocol was implemented. This may have led to variations in clinical practice for patients with primary PPH throughout the study. However, it is important to note that the activation criteria of the protocol only consider hemodynamic instability and the anticipated volume of RBC transfusion, without taking into account the coagulation status determined by TEG or fibrinogen levels. Fourth, we only used the threshold for hypofibrinogenemia, defined as a fibrinogen level < 200 mg/dL. If we adopted a different threshold with clinical significance, the results would be different. Fifth, we only used the initial TEG value to predict hypofibrinogenemia. Given the dynamic nature of the clinical manifestations of PPH, it would be more beneficial to have data on the serial follow-up of TEG values. Finally, it is possible that the optimal timing for implementing TEG may not coincide with the time of admission. Further studies are needed to clarify the results of future investigations.

## Conclusion

This study revealed that point-of-care TEG could be a valuable tool for the early identification of hypofibrinogenemia in ED patients with primary PPH. Measuring TEG upon ED admission, along with other objective parameters, can help rapidly stratify patients based on their risk and guide early interventions, such as transfusion strategies.

## Data Availability

The datasets used and analyzed during the current study are available from the corresponding author upon reasonable request.

## Abbreviations:

AUC: Area under the curve
AVPU: Alert/verbal/painful/unresponsive
CI: Confidence interval
ED: Emergency department
ICU: Intensive care unit
IQR: Interquartile range
K: Kinetic time
LY30: Lysis after 30 min
MA: Maximum amplitude
MT: Massive transfusion
NPV: Negative predictive value
OR: Odds ratio
PPH: Postpartum hemorrhage
PPV: Positive predictive value
R: Reaction time
ROC: Receiver operating characteristic
TEG: Thromboelastography
